# The XPO1 Inhibitor KPT-8602 Ameliorates Parkinson’s Disease by Inhibiting the NF-κB/NLRP3 Pathway

**DOI:** 10.3389/fphar.2022.847605

**Published:** 2022-06-01

**Authors:** Shuhan Liu, Shengxiang Wang, Runze Gu, Na Che, Jing Wang, Jinbo Cheng, Zengqiang Yuan, Yong Cheng, Yajin Liao

**Affiliations:** ^1^ Center on Translational Neuroscience, College of Life and Environmental Sciences, Minzu University of China, Beijing, China; ^2^ Key Laboratory of Ecology and Environment in Minority Areas (Minzu University of China), National Ethnic Affairs Commission, Beijing, China; ^3^ The Brain Science Center, Beijing Institute of Basic Medical Sciences, Beijing, China

**Keywords:** XPO1, KPT-8602, Parkinson’s disease, NLRP3 inflammasome, NF-κB

## Abstract

Exportin 1 (XPO1) is an important transport receptor that mediates the nuclear export of various proteins and RNA. KPT-8602 is a second-generation inhibitor of XPO1, demonstrating the lowest level of side effects, and is currently in clinical trials for the treatment of cancers. Previous studies suggest that several first-generation inhibitors of XPO1 demonstrate anti-inflammation activities, indicating the application of this drug in inflammation-related diseases. In this study, our results suggested the potent anti-inflammatory effect of KPT-8602 *in vitro* and *in vivo*. KPT-8602 inhibited the activation of the NF-κB pathway by blocking the phosphorylation and degradation of IκBα, and the priming of NLRP3. Importantly, the administration of KPT-8602 attenuated both lipopolysaccharide (LPS)-induced peripheral inflammation and 1-methyl-4-phenyl-1,2,3,6-tetrahydropyridine (MPTP)-induced neuroinflammation *in vivo*. In addition, the tissue damage was also ameliorated by KPT-8602, indicating that KPT-8602 could be used as a novel potential therapeutic agent for the treatment of inflammasome-related diseases such as Parkinson’s disease, through the regulation of the NF-κB signaling pathway and the NLRP3 inflammasome.

## Introduction

Exportin 1 (XPO1), also referred to as chromosomal maintenance region 1 (CRM1), is a key nuclear transport receptor involved in the export of more than 200 known cargo proteins, including tumor suppressors, anti-inflammatory factors, and growth-regulating proteins (Hutten and Kehlenbach, 2007; Xu et al., 2010; Xu et al., 2012). XPO1 mediates the nuclear transport process by specifically recognizing the leucine-rich nuclear export signal (NES) in cargo proteins (Hutten and Kehlenbach, 2007). Previous studies suggest that XPO1 is involved in the transport of proteins related to oncogenesis, including p53, p73, FOXO, PI3K/AKT, and Wnt/β-catenin, indicating its pivotal regulatory role in cancer therapy (Kau et al., 2004; Turner et al., 2012; Hill et al., 2014). Also, the upregulation of XOP1 causes alterations in the process of cell apoptosis, DNA damage repair, chromosome stability, and angiogenesis (Sun et al., 2016; Wang and Liu, 2019). Therefore, XPO1 is considered an effective target for the treatment of cancer, inflammation, and autoimmune diseases, through the regulation of nuclear–cytoplasmic localization of important proteins (Mao and Yang, 2013; Gravina et al., 2014; Haines et al., 2015; Olazagoitia-Garmendia et al., 2021).

**FIGURE 1:**
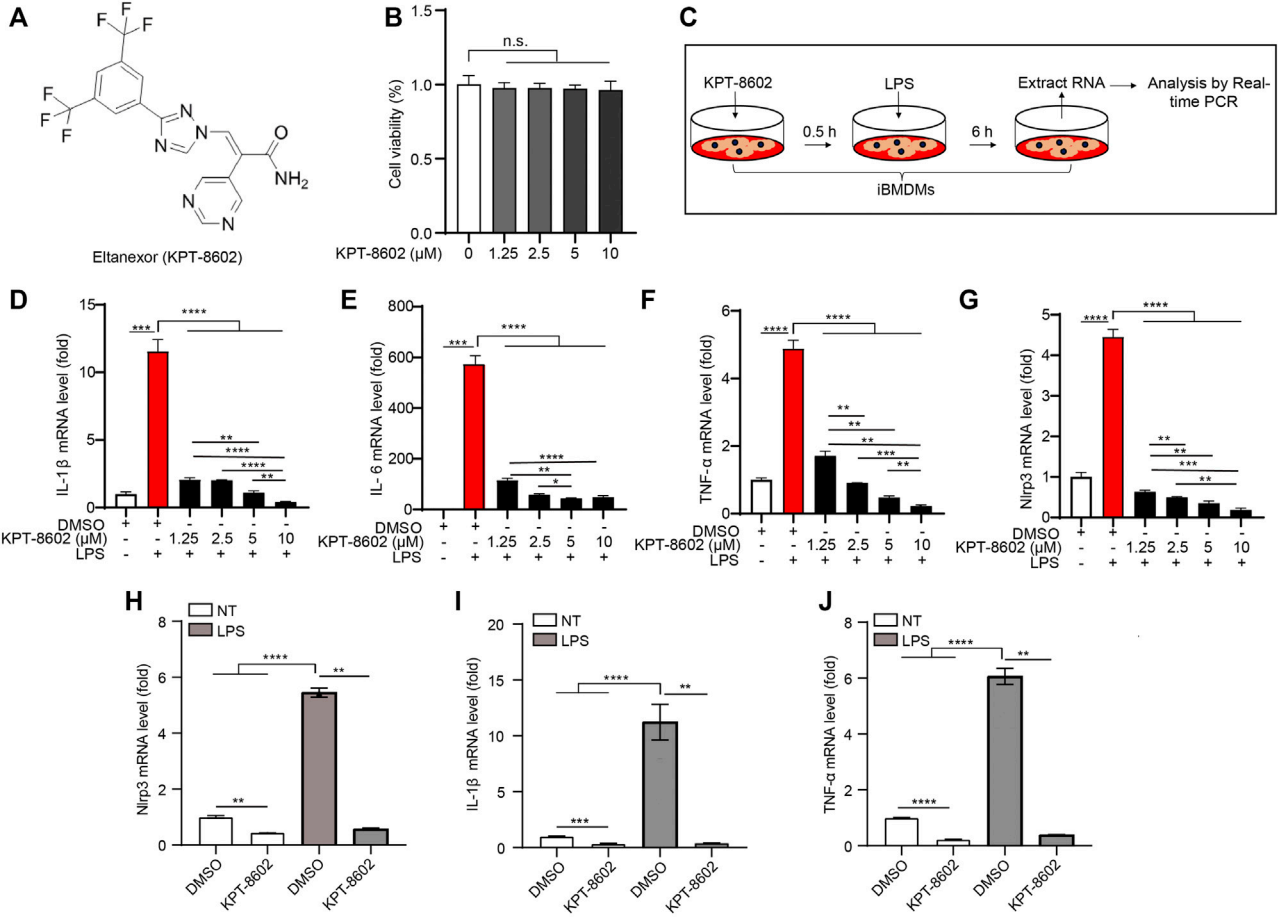
KPT-8602 inhibits LPS-induced high expression of pro-inflammatory cytokines in macrophages. **(A)** Structure of KPT-8602. **(B)** iBMDMs were treated with various doses of KPT-8602 for 6 h, and cell viability was assessed by the CCK-8 assay kit. **(C)** Working model of LPS-induced inflammation. **(D–G)** iBMDMs were pretreated with KPT-8602 at different concentrations and stimulated with LPS for 6 h, and then the mRNA levels of IL-1β **(D)**, IL-6 **(E)**, TNF-α **(F)**, and Nlrp3 **(G)** were analyzed by real-time PCR. **(H–J)** Primary peritoneal macrophages (PMs) pretreated with KPT-8602 (5 μM) were treated with LPS for 6 h, and then the mRNA levels of Nlrp3 **(H)**, IL-1β **(I)**, and TNF-α **(J)** were analyzed by real-time PCR. (^**^ indicates *p* < 0.01, ^***^ indicates *p* < 0.001, ^****^ indicates *p* < 0.0001 by one-way ANOVA). n. s. no significant, NT, no treatment.

In recent years, several small-molecule selective inhibitors of nuclear export (SINE) compounds with anticancer activities are discovered. Selinexor (also called KPT-330), is a first-in-class oral SINE compound that has been shown to induce nuclear aggregation of tumor suppressor proteins and demonstrate anticancer activities in preclinical and clinical research and is currently being evaluated in phase I/II/III clinical trials for its potential use in hematologic and solid tumors (Lang et al., 2019; Taylor-Kashton et al., 2016; Azmi et al., 2021). However, the presence of its systemic toxicities and brain/blood-related adverse reactions limited its clinical application due to safety reasons (Jakubowiak et al., 2019; Chen et al., 2018). Eltanexor (also called KPT-8602, Figure 1A) is a second-generation SINE compound developed by Karyopharm to address the high toxicity of selinexor, and it has been shown to have similar efficacy to or even better efficacy than selinexor in hematological malignancies in animal models (Lang et al., 2019). Also, due to its low central nervous system (CNS) penetration rate, eltanexor is therefore better tolerated and can be used in a wider therapeutic window (Hing et al., 2016; Etchin et al., 2017).

The NOD-like receptor protein 3 (NLRP3) inflammasome is one of the major inflammasomes activated by a variety of pathogen-associated molecules and damage-associated molecules (Pan et al., 2021). The activation of the NLRP3 inflammasome is regulated by a 2-step activation process including the priming phase, characterized by the activation of the nuclear factor kappa B (NF-κB) pathway that promotes the expression of NLRP3 and the precursor of IL-1β; and the activation phase, featured by the assembly of the NLRP3 inflammasome induced by stimuli such as nigericin (Nig) (Elliott and Sutterwala, 2015; Shao et al., 2015). Our previous study reveals that aberrant activation of the NLRP3 inflammasome contributes to neurodegenerations and development of Parkinson’s disease (PD) through the promotion of inflammation and induction of pyroptosis (Cheng et al., 2020).

In this study, we demonstrated that KPT-8602 suppressed the activation of the NF-κB signaling pathway by inhibiting the phosphorylation and degradation of IκBα, and therefore inhibited the transcription of NLRP3. Moreover, *in vivo* study revealed that lipopolysaccharide (LPS)-induced peripheral inflammation and 1-methyl-4-phenyl-1,2,3,6-tetrahydropyridine (MPTP)-induced neuroinflammation were both downregulated by the administration of KPT-8602. Consistently, the tissue injury was also ameliorated by administration of KPT-8602, suggesting that KPT-8602 is a potential therapeutic agent for the treatment of diseases associated with aberrant activation of the NF-κB signaling pathway and the NLRP3 inflammasome.

## Materials and Methods

### Mice

C57BL/6 mice were purchased from Vital River Laboratory Animal Technology Co., Ltd (Beijing). For the experiment, four to five mice were housed per cage under a 12-h light/dark cycle at 22–24°C and given unrestricted access to food and water. All the animal experimental procedures were approved by the Institutional Animal Care and Use Committee of the Beijing Institute of Basic Medical Sciences.

### Cell Culture and Treatment

Immortalized murine bone marrow-derived macrophages (iBMDMs) or BV2 microglial cell lines were maintained in Dulbecco s modified Eagle s medium (#11965–092, Life Technologies, Waltham, MA, United States ) supplemented with 10% heat-inactivated fetal bovine serum (FBS, #04-001-1A, Biological Industries, Beit Haemek, Israel) and 1% penicillin–streptomycin solution (#03-031-1B, Biological Industries) at 37°C in a humidified atmosphere with 5% CO_2_. For LPS-induced inflammation *in vitro*, iBMDMs were pretreated with 1.25, 2.5, 5, and 10 μM of KPT-8602 for 30 min, and stimulated with LPS (1 μg/ml) for 6 h.

### Cell Viability Assay

A Cell Counting Kit-8 (CCK-8) assay (ab228554; Abcam, Cambridge, UK) was used to evaluate cell viability following the manufacturer’s instructions. Briefly, iBMDMs were plated at a density of 1 × 10^5^ cells/mL in 96-well plates and exposed to different concentrations of KPT-8602 for 6 h. Subsequently, 10 μL of CCK-8 reagent was added to each well and incubated at 37°C for an additional 2 h. Finally, a Spectra Max i3x (Molecular Devices, Sunnyvale, CA, United States) was used to measure the absorbance at 450 nm.

### Isolation and Culture of Primary Macrophages

Primary peritoneal macrophages (PMs) from 8-week-old wild-type mice were isolated and cultured as previously described (Pan et al., 2021). Briefly, the mice were killed by using the cervical dislocation method and 75% ethyl alcohol disinfection for 5–10 min. PMs were collected after washing the peritoneal cavity with 5 ml of an ice-cold serum-free RPMI-1640 medium (#C11875500BT, Gibco, Shanghai, China). After that, the PMs were centrifuged at 300 *g* and 4°C for 5– min, and the cell pellets were resuspended in a fresh RPMI-1640 medium (supplemented with 10% heat-inactivated FBS, 1% penicillin, and 1% streptomycin) and then seeded in culture plates for subsequent experiments.

### SiRNA-Mediated Gene Silencing in iBMDMs and PMs

iBMDMs/PMs (3 × 10^5^ cells/mL) were plated in 12-well plates and siRNA (50 nM) was transfected into the cells in each well using Lipofectamine RNAiMAX (Invitrogen) as per the manufacturer’s instructions. The siRNA scramble and siRNA against *XPO1* were obtained from Genepharma (Suzhou, China).

### Western Blotting

Cells or tissues were lysed with RIPA lysis buffer comprising a cocktail of protease and phosphatase inhibitors, and total protein concentrations were measured by BCA assay and boiled at 100°C for 15 min. The equal amounts of proteins were separated by SDS-PAGE gel at 80 V for 0.5 h and 120 V for 1 h. After that, the protein was transferred to a PVDF membrane (#ISEQ00010, Millipore, Darmstadt, Hessen, Germany) at 250 mA for 1.5 h. The membrane was blocked for 1 h at room temperature using blocking buffer (5% nonfat milk) and was then incubated with primary antibodies overnight at 4°C. The next morning, the membrane was washed with Tris-buffered saline and Tween-20 (TBST) three times for 5 min each, followed by incubated with conjugated secondary antibodies for protein detection. The primary antibodies used in the present study are as follows: anti-phospho-IKKα/β (#2697P, Cell Signaling Technology, MA, United States, 1:1000), anti-phospho-IκBα (#2859, Cell Signaling Technology, 1:1000), anti-IKKα (#A2062, ABclonal Technology, Wuhan, HB, China, 1:1000), anti-IκBα (#4814, Cell Signaling Technology, 1:1000), anti-exportin 1/CRM1 (#46249, Cell Signaling Technology, 1:1000), anti-GAPDH (CW0266A, CWBiotech, Beijing, China, 1:2000), anti-H2B (ab64165, Abcam, 1:1000), anti-phospho-NF-κB p65 (#3033, Cell Signaling Technology, 1:1000), anti-NF-κB p65 (#8242, Cell Signaling Technology, 1:1000 for Western blotting, 1:400 for immunofluorescence), anti-NLRP3 (#AG-20B-0014, AdipoGen, San Diego, CA, United States, 1:1000), anti-caspase-1 (#Ag-20B-0042, AdipoGen, 1:1000), anti-IL-1β (#AF-401-NA, R&D Systems, Minneapolis, MN, United States, 1:1000), anti-ASC (#67824, Cell Signaling Technology, 1:1000 for Western blotting, 1:200 for immunofluorescence), anti-TH (2792, Cell Signaling Technology, 1:1000 for Western blotting, 1:400 for immunochemistry), anti-Iba1 (ab5076, Abcam, 1:1000 for Western blotting, 1:400 for immunofluorescence), anti-β-actin (60008-1-Ig, Proteintech Group, Campbell Park, Chicago, IL, United States, 1:2000 for Western blotting, 1:500 for immunofluorescence), and anti-β-tubulin (#CW0098A, CWBiotech, Taizhou, JS, China, 1:2000 for Western blotting, 1:500 for immunofluorescence).

### Enzyme-Linked Immunosorbent Assay

PMs treated with LPS (1 μg/ml) for 3.5 h were further stimulated with Nig for 45 min. Then, the supernatants were collected and centrifuged at 12,000 × *g* and 4°C for 5 min. The concentration of IL-1β in the supernatants (#432604, BioLegend, San Diego, CA, United States) was determined by ELISA following the manufacturer’s instructions.

### Assessment of LPS-Induced Systemic Inflammation

Eight-week-old C57BL/6 male mice (body weight: 22–25 g) were orally administered with 5 mg/kg KPT-8602 or vehicle (saline) and then intraperitoneally injected with LPS (10 mg/kg). After 4 h, all animals were killed, and tissue samples from the liver, lung, and kidney were collected for subsequent experiments.

### Histological Analysis

Tissue samples from the liver, lung, and kidney were fixed with 4% paraformaldehyde (PFA) in 0.1 M phosphate buffer (pH 7.4) for 24 h, and 5 μm coronal paraffinized sections were prepared for histological assessment. The sections of various organs were stained with hematoxylin and eosin (H&E). The pathological scores for these organs were determined as described in previous studies with minor modifications (Maehara et al., 2020) and were mainly determined as the degree of immune cell infiltration and structure disruption, with a scale of 0–3 as follows: 0 = none, 1 = mild, 2 = moderate, and 3 = severe.

### Induction and Assessment of MPTP-Induced PD

Eight-week-old C57BL/6 male mice (body weight: 22–25 g) were orally administered 5 mg/kg KPT-8602 or vehicle (saline) for 6 days at 12 h after the MPTP injection (Figure 6A). The mice were administered four intraperitoneal injections of 20 mg/kg MPTP as previously described (Wu et al., 2016). At 7 days after the final MPTP injection, all animals were killed, and the substantia nigra and striatum tissue samples of one of the cerebral hemispheres of mice in each group were collected for the Western blot analysis. The other hemispheres were infused with 4% paraformaldehyde in 0.1 M phosphate buffer (pH 7.4), and 40 *μ*m coronal frozen sections were prepared for immunohistochemical assays.

### Behavioral Tests

For the rotarod test, the motor capacity of the mice was assessed using a rotarod apparatus (Panlab, Barcelona, Spain, LE8200). Briefly, the mice were placed on a rotation rod, with the rotation speed gradually increased from 4 to 40 rpm over a period of 5 min, and the latency to falling was recorded. Three tests were performed at an interval of 1 h, and the average of the three tests was taken as the final test result. The mice were acclimated to the environment prior to each training and test session.

### Immunochemistry

All animals were euthanized with tribromoethanol, then precooled normal saline was infused into the heart and brain tissue was fixed with 4% PFA for at least 24 h. The coronal sections were then immunochemically treated with anti-TH antibody staining and confocal analysis. For TH labeling, briefly, the slices were incubated with rabbit polyclonal anti-TH antibodies (1:400, Cell Signaling Technology) and visualized with biotinylated goat anti-rabbit IgG, followed by streptavidin-conjugated horseradish peroxidase (Vectastain ABC kit, Zhongshanjinqiao, Beijing, China). Positive immunostaining was visualized with 3,3-diaminobenzidine (DAB) peroxidase substrate (DAB kit, Vector Laboratories). Stained sections were mounted onto slides and analyzed by Stereo Investigator software (MicroBrightfield, Williston, VT, United States).

### Stereological Analysis

All procedures were performed as previously described (Zheng et al., 2021). Briefly, 40-*μ*m coronal sections were cut throughout the brain, including the substantia nigra and striatum, and every fourth section was used for analysis by Stereo Investigator software.

### Multiplex Immunofluorescence Staining

The tyramide signal amplification (TSA)-based Opal staining method was used to stain multiple markers from the same species of SNc sections as described in the previous studies and modified (Fan et al., 2021). Briefly, tissue sections were placed in citrate buffer (10 mM sodium citrate, 0.05% Tween 20, pH 6.0) for 30 min at a sub-boiling temperature, washed twice in PBS, and then blocked in 3% hydrogen peroxide for 20 min at room temperature. The samples were then washed thrice in PBS, blocked in blocking solution (5% fetal bovine serum, 0.5% Triton X-100, 1% bovine serum albumin) for 1 h at room temperature, incubated with anti-rabbit Iba1 antibody (1:400, Abcam) at 4°C for 30 min, and then incubated at room temperature for 30min, washed in PBST (PBS and Tween 20, pH 7.6), incubated in poly-HRP-conjugated secondary antibody, and washed again in PBST before incubation in a tyramide working solution (e.g., AlexaFluor 488 tyramide) for 10 min followed by immediate application of reaction stop reagent working solution. For the second round of staining, tissue sections were first rinsed three times in PBST before being placed in citrate buffer for 30 min and blocked in 3% hydrogen peroxide and blocking solution again before application of anti-rabbit ASC (1:200, CST) and secondary antibody, followed by treatment with a second tyramide working solution (e.g., AlexaFluor 594 tyramide). For TH marker staining, the process was repeated once more with a third tyramide working solution (e.g., AlexaFluor 647 tyramide). Tissue sections were re-stained with DAPI, covered with slides (CITOGLAS, 188105W) and then dried overnight at 4°C before confocal microscopic imaging.

The number of ASC speck-positive cells was analyzed by the formula as follows: Ratio = the number of ASC speck-positive cells/the number of total cells. The number of Iba-1-positive cells was counted in the same way.

### ASC Oligomerization Assay

The ASC oligomerization assay was performed as previously reported (Pan et al., 2021). Briefly, 45 min post-Nig stimulation, primary macrophages were rinsed in ice-cold PBS and then lysed with hypotonic lysis buffer (10 mM KCl, 1.5 mM MgCl_2_, 1 mM EDTA, 1 mM EGTA, 0.1mM PMSF, and 20 mM Tris; pH 7.5) and incubated on ice for 30 min, shaking every 5 min. The lysates were centrifuged at 6,000 × *g* for 8 min at 4°C, and then the pellets were washed three times in ice-cold PBS and resuspended in 500 μL CHAPS buffer (0.1% CHAPS, 10 mM KCl, 1.5 mM MgCl_2_, 1 mM EDTA, 1 mM EGTA, 0.1 mM PMSF, and 20 mM Tris; pH 7.5). The resuspended pellets were incubated with disuccinimidyl suberate (DSS, #S1885, Sigma-Aldrich, 2 mM) for 45 min at 37°C with rotation. The samples were then centrifuged at 6, 000 × *g* for 15 min at 4°C. The cross-linked pellets were resuspended in 60 μL of sample buffer and were then analyzed by Western blotting.

### Dual-Luciferase Reporter System

The NF-κB reporter was generated in our laboratory (Li et al., 2020). Briefly, the NF-κB promoter was cloned into a pGL3-luciferase reporter vector (Promega, Madison, United States). Then, HEK293T cells were co-transfected with the pCMV-Renilla plasmid and NF-κB reporter using Lipofectamine 2000 transfection reagent (#11668019, Invitrogen). The cells were lysed 24 hours after transfection, and luciferase activity was measured using a dual-luciferase reporter detection system (Promega).

### Real-Time Quantitative Polymerase Chain Reaction

Total RNA was isolated from the cells or tissues using TRIzol reagent (#15596026, Invitrogen), and RNA (1 ug) from each sample was used for reverse transcription with a One-Step First-strand cDNA synthesis kit (#AT311-02, Transgen, Beijing, China). SYBR Green-based real-time qPCR (#A304, GenStar, Beijing, China) was used to measure target gene expression. The sequences of the gene-specific primers used are as follows:

mouse *Il1b* forward, GTC​GCT​CAG​GGT​CAC​AAG​AA.
mouse *Il1b* reverse, CTG​CTG​CCT​AAT​GTC​CCC​TT.
mouse *Il6* forward, GCT​ACC​AAA​CTG​GAT​ATA​ATC​AGG​A.
mouse Il6 reverse, CCA​GGT​AGC​TAT​GGT​ACT​CCA​GAA.
mouse Tnf forward, CAG​GCG​GTG​CCT​ATG​TCT​C.
mouse Tnf reverse, CGA​TCA​CCC​CGA​AGT​TCA​GTA​G;
mouse Inos forward, GTT​CTC​AGC​CCA​ACA​ATA​CAA​GA.
mouse Inos reverse, GTG​GAC​GGG​TCG​ATG​TCA​C;
mouse Nlrp3 forward, ATT​ACC​CGC​CCG​AGA​AAG​G.
mouse Nlrp3 reverse, TCG​CAG​CAA​AGA​TCC​ACA​CAG.
mouse *β*-actin forward, GGT​GAA​GGT​CGG​TGT​GAA​CG.
mouse *β*-actin reverse, CTC​GCT​CCT​GGA​AGA​TGG​TG.

### Statistical Analysis

The gray values of the Western blot bands were analyzed by ImageJ software (NIH, Bethesda, MD, United States). All the data represent three independent repeat experiments and the significance was performed with the *t*-test for two groups, or one-way ANOVA for multiple groups (GraphPad Software, San Diego, CA, United States). All values are expressed as the mean ± S.E.M. Differences between data were considered significant when the *p* value was <0.05.

## Results

### KPT-8602 Inhibits LPS-Induced Inflammation *In Vitro*

To test the potential cytotoxicity of KPT-8602 on cells, we treated iBMDMs with a serial dose of KPT-8602. The results showed that KPT-8602 used the following10 uM displayed no obvious toxic effects on iBMDMs (Figure 1B). Then, iBMDMs were pretreated with 1.25, 2.5, 5, and 10 μM of KPT-8602 for 30 min and followed by stimulating with LPS (1 μg/ml) for 6 h (Figure 1C), and we found that the expression of IL-1β (Figure 1D), IL-6 (Figure 1E), and TNF-α (Figure 1F) induced by LPS were inhibited by KPT-8602. In addition, LPS-induced upregulation of NLRP3 was also blocked by KPT-8602 (Figure 1G), suggesting its inhibitory effect on the transcription of NLRP3. These results indicated that 5 μM KPT-8602 was sufficient to inhibit the expression of pro-inflammatory cytokines and NLRP3, with no obvious cytotoxicity in iBMDMs. In addition, LPS-induced transcription of NLRP3 (Figure 1H), IL-1β (Figure 1I), and TNF-α (Figure 1J) were also significantly inhibited by KPT8602 at 5 μM in PMs. Therefore, the concentration of KPT-8602 used in the subsequent study was five uM. Taken together, these results suggested that KPT-8602 effectively inhibited LPS-induced inflammation *in vitro*.

### KPT-8602 Inhibits the Activation of the NLRP3 Inflammasome in Primary Macrophage

**FIGURE 2:**
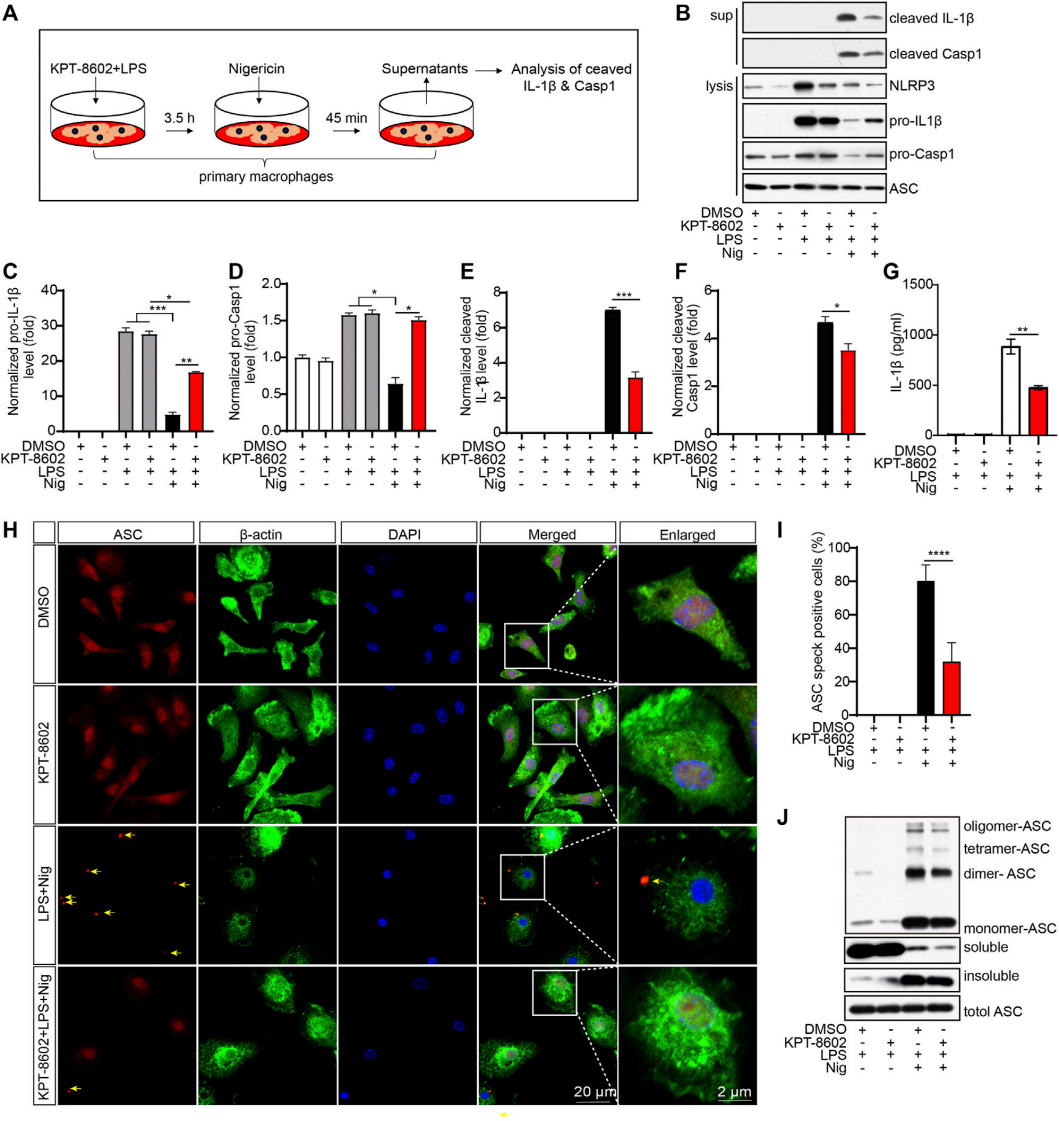
KPT-8602 inhibits the activation and assembly of the NLRP3 inflammasome *in vitro*. **(A)** Working model of the NLRP3 inflammasome activation. **(B–F)** PMs pretreated with LPS were treated with KPT-8602 for 30 min and nigericin (Nig) for 45 min, and the cells and supernatants were harvested for analysis of the expression of NLRP3, pro-IL-1β, pro-caspase-1, cleaved IL-1β, cleaved caspase-1, and ASC by Western blotting **(B)**. Gray values of the pro-IL-1β **(C)**, pro-Casp-1 **(D)**, cleaved IL-1β **(E)**, and cleaved Casp-1 **(F)** bands were analyzed by using ImageJ and were normalized to ASC. **(G)** PMs pretreated with LPS were treated with KPT-8602 for 30 min and nigericin (Nig) for 45 min, and the cells and supernatants were harvested for analysis of the expression of cleaved IL-1β by ELISA. **(H,I)** PMs pretreated with LPS were treated with KPT-8602 for 30 min and nigericin (Nig) for 45 min. Then, the cells were fixed and stained with a rabbit anti-ASC antibody and mouse anti-β-actin antibody **(H)**, and the number of cells containing ASC specks (yellow arrows) was analyzed by ImageJ. **(I)** Scale bars, 20 μm for low-magnification images and 2 μm for high-magnification images, respectively. **(J)** PMs pretreated with LPS were treated with KPT-8602 and Nig for 45 min, and then the cells were harvested and cross-linked with disuccinimidyl suberate (DSS) for analysis of the oligomerization of ASC by Western blotting. (^*^ indicates *p* < 0.05, ^**^ indicates *p* < 0.01, ^***^ indicates *p* < 0.001, ^****^ indicates *p* < 0.0001 by Student’s t test or one-way ANOVA).

As KPT-8602 inhibited the transcription of NLRP3 and IL-1β, we next analyzed the effects of KPT8602 on the activation of the NLRP3 inflammasome (Figure 2A). The results displayed that the cleavage of caspase-1 and IL-1β induced by LPS and Nig were significantly reduced by KPT-8602 in PMs (Figures 2B–F). Consistently, the concentration of secreted IL-1β in the supernatants was also decreased in KPT-8602-treated PMs (Figure 2G). We then analyzed the effects of KPT-8602 on the assembly of the NLRP3 inflammasome and found that the number of ASC specks induced by LPS and Nig were significantly reduced by KPT-8602 (Figures 2H, I). In addition, the formation of ASC dimers, tetramers, and oligomers was significantly reduced by KPT-8602 as well (Figure 2J; Supplementary Figure S1). Together, these results indicate that KPT-8602 inhibited the activation of the NLRP3 inflammasome *in vitro*.

### KPT-8602 Exerts Anti-Inflammatory Effects by Blocking the NF-κB Pathway

**FIGURE 3:**
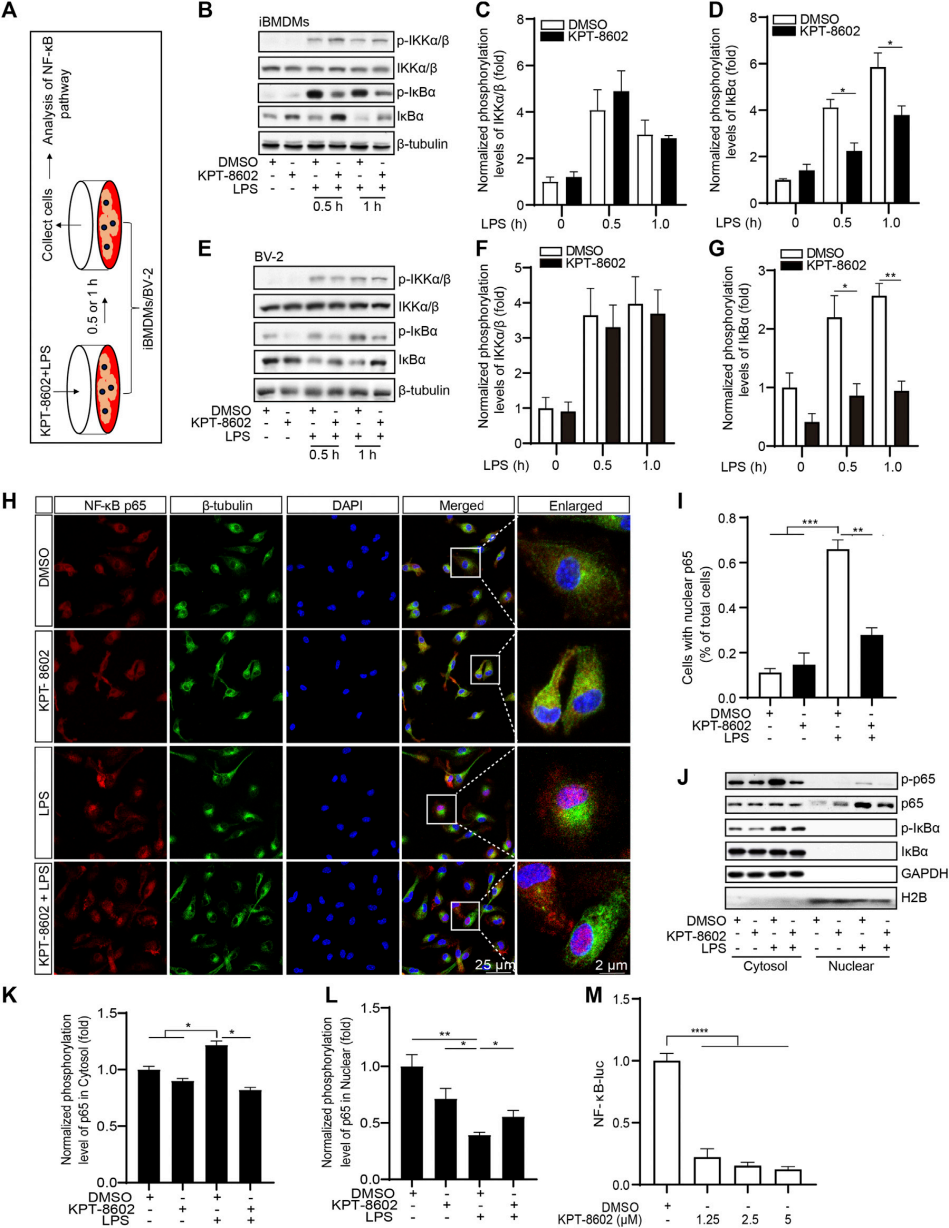
KPT-8602 inhibited LPS-induced activation of the NF-κB signaling pathway. **(A)** iBMDMs or BV-2 were pretreated with or without KPT-8602 (5 μM) and stimulated with LPS for 0.5 and 1 h, respectively, and then the cell lysates were collected for analysis by Western blotting **(B,E)**. The gray values of the phosphorylated and total IKKα/β **(C,F)** and IκBα **(D,G)** bands were analyzed with ImageJ. **(H,I)** PMs were pretreated with KPT-8602 for 30 min and stimulated with LPS for 2 h. Then, the cells were fixed and stained with a rabbit anti-NF-κB p65 antibody and mouse anti-β-tubulin antibody **(H)**, and the number of cells containing nuclear p65 was analyzed by ImageJ **(I)**. Scale bars, 25 μm for low-magnification images and 2 μm for high-magnification images, respectively. **(J,K)** iBMDMs were pretreated with KPT-8602 for 1 h and stimulated with LPS for 2 h, and then extracts from the nucleus and cytoplasm were collected for analysis by Western blotting **(J)**. The gray value of the phosphorylated and total p65 in the nucleus **(K)** and cytoplasm **(L)** were analyzed with ImageJ. H2B served as a nuclear protein marker; GAPDH as a cytosolic protein marker. **(M)** Quantitative analysis of the effect of KPT-8602 on NF-κB luciferase activity. (^*^ indicates *p* < 0.05, ^**^ indicates *p* < 0.01, ^***^ indicates *p* < 0.001, ^****^ indicates *p* < 0.0001 by one-way ANOVA).

To further analyze the anti-inflammatory mechanism of KPT-8602, we next investigated the role of KPT-8602 on the activation of the NF-κB signaling pathway (Figure 3A). The results revealed that KPT-8602 had no effect on the LPS-induced phosphorylation of IKKα/β. However, we found KPT-8602 significantly inhibited IKKα/β-mediated phosphorylation of IκBα in iBMDMs (Figures 3B–D). Consistently, the phosphorylation levels of IκBα induced by LPS in BV2 microglial cells were also inhibited by KPT-8602 (Figures 3E–G). The reduced phosphorylation of IκBα caused decreased degradation of IκBα (Figures 3B, E) and decreased nuclear translocation p65 in KPT-8602-treated PMs (Figures 3H, I) and iBMDMs (Figure 3J). In addition, the LPS-induced phosphorylation of p65 was blocked by KPT-8602 (Figure 3K–l). Furthermore, the NF-κB response element reporter activity was also impaired in KPT-8602-treated HEK293T cells (Figure 3M).

**FIGURE 4:**
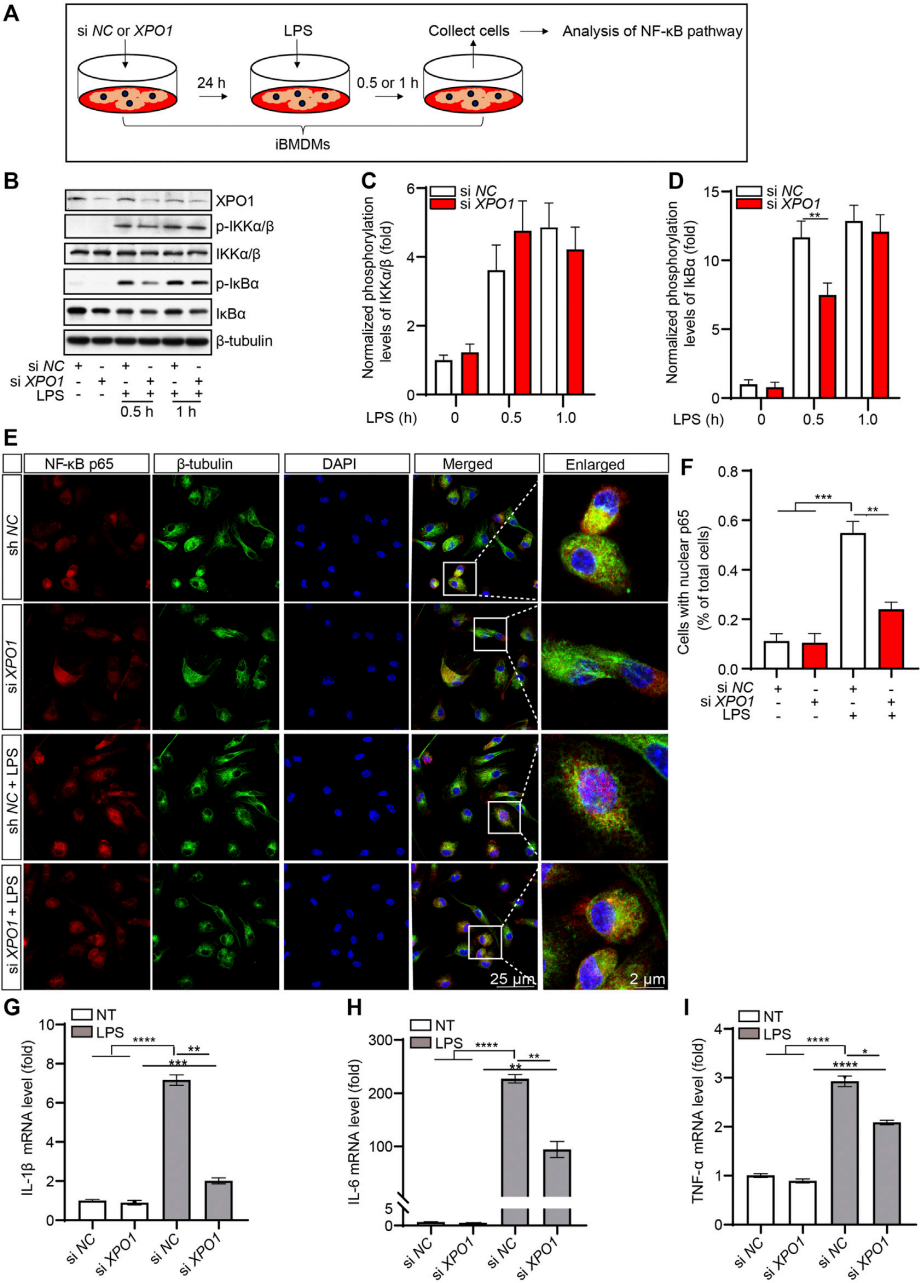
Silencing XPO1 inhibits NF-κB signaling pathway and exerts anti-inflammatory activity. **(A)** iBMDMs transfected with siRNA against XPO1 or scrambled siRNA were treated with LPS for 0.5 and 1 h, respectively, and then the cell lysates were collected for analysis by Western blotting **(B)**. The gray values of the phosphorylated and total IKKα/β **(C)** and IκBα **(D)** bands were analyzed with ImageJ. **(E,F)** PMs transfected with siRNA against XPO1 or scrambled siRNA were stimulated with LPS for 2 h, and then the cells were fixed and stained with a rabbit anti-NF-κB p65 antibody and mouse anti-β-tubulin antibody **(E)**, and the number of cells containing nuclear p65 was analyzed by ImageJ **(F)**. Scale bars, 25 μm for low-magnification images and 2 μm for high-magnification images, respectively. **(G–I)** iBMDMs transfected with siRNA against XPO1 or scrambled siRNA were treated with LPS for 6 h, and then the mRNA levels of IL-1β **(G)**, IL-6 **(H)**, and TNF-α **(I)** were detected by real-time PCR. (^*^ indicates *p* < 0.05, ^**^ indicates *p* < 0.01, ^***^ indicates *p* < 0.001, ^****^ indicates *p* < 0.0001 by one-way ANOVA).

To investigate whether the knockdown of XPO1 has the same effect on the activation of the NF-κB pathway, we generated XPO1-silenced iBMDMs (Figure 4A). As suggested by the results, LPS-induced phosphorylation of IκBα was inhibited in XPO1-silenced iBMDMs. Meanwhile, no significant alteration was observed in the phosphorylation levels of IKKα/β (Figures 4B–D). We also found that the nuclear translocation of p65 induced by LPS was also impaired in XPO1-silenced PMs (Figures 4E, F). Moreover, LPS-induced transcription of IL-1β (Figure 4G), IL-6 (Figure 4H), and TNF-α (Figure 4I) was inhibited by silencing of XPO1. Taken together, the aforementioned results suggested that KPT-8602 inhibits the activation of the NF-κB pathway by blocking the phosphorylation of IκBα.

### KPT-8602 Suppresses LPS-Induced Systemic Inflammation *In Vivo*

**FIGURE 5:**
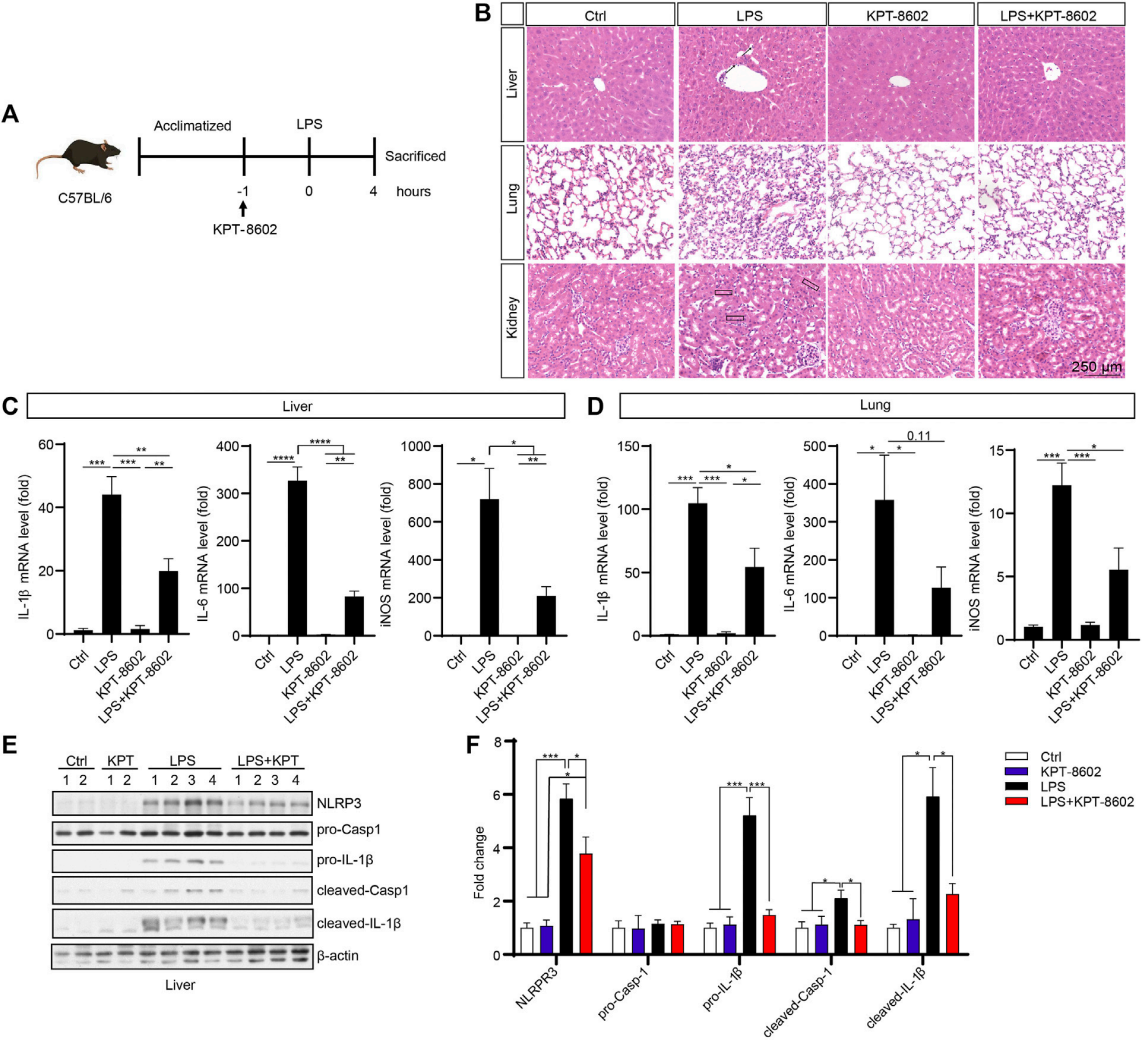
KPT-8602 suppresses LPS-induced systemic inflammation. **(A)** C57BL/6 mice were given LPS (10 mg/kg) intraperitoneally after oral admiration of KPT-8602 (5 mg/kg) or vehicle (5 mice per group). **(B)** Representative photomicrographs of paraffin-embedded sections of the liver, lung, and kidney tissues stained with H&E. Scale bars, 250 μm for images, respectively. The expression levels of IL-1β, IL-6, and iNOS in the liver **(C)** and lung **(D)** were detected by real-time PCR. The expression of NLRP3, pro-caspase-1, pro-IL-1β, cleaved caspase-1, cleaved-IL-1β, and *β*-actin in the liver was analyzed by Western blotting **(E)**, and the gray values of their bands were analyzed by using ImageJ and were normalized to *β*-actin **(F)**. (^*^ indicates *p* < 0.05, ^**^ indicates *p* < 0.01, ^***^ indicates *p* < 0.001, ^****^ indicates *p* < 0.0001 by one-way ANOVA).

We further evaluated the potential anti-inflammatory effect of KPT-8602 *in vivo* in an LPS-induced inflammation mice model (Figure 5A). We found that the LPS-induced macrophage infiltration (indicated by arrow) in the liver (Figure 5B; Supplementary Figure.3A), structural destruction (indicated by arrowheads) of the lung (Figure 5B; Supplementary figure.3B), and the kidney (Figure 5B; Supplementary Figure 3C) were attenuated by administration of KPT-8602. In addition, the LPS-induced expression of pro-inflammatory cytokines, such as IL-1β, IL-6, and iNOS, in the liver (Figure 5C) and lung (Figure 5D) were downregulated by KPT-8602 administration. Furthermore, the expression of IL-1β precursor and NLRP3, and mature IL-1β (cleaved IL-1β) in the liver was significantly inhibited by KPT-8602 (Figures 5E, F). Consistently, the level of mature IL-1β and expression of NLRP3 induced by LPS was also decreased in the lung in KPT-8602-treated mice (Supplementary Figures. 3D, E). Together, these results suggested that KPT-8602 inhibited the activation of the NF-κB pathway and the NLRP3 inflammasome *in vivo*.

### KPT-8602 Ameliorates MPTP-Induced Dopaminergic Neuron Loss and Microglial Activation

**FIGURE 6:**
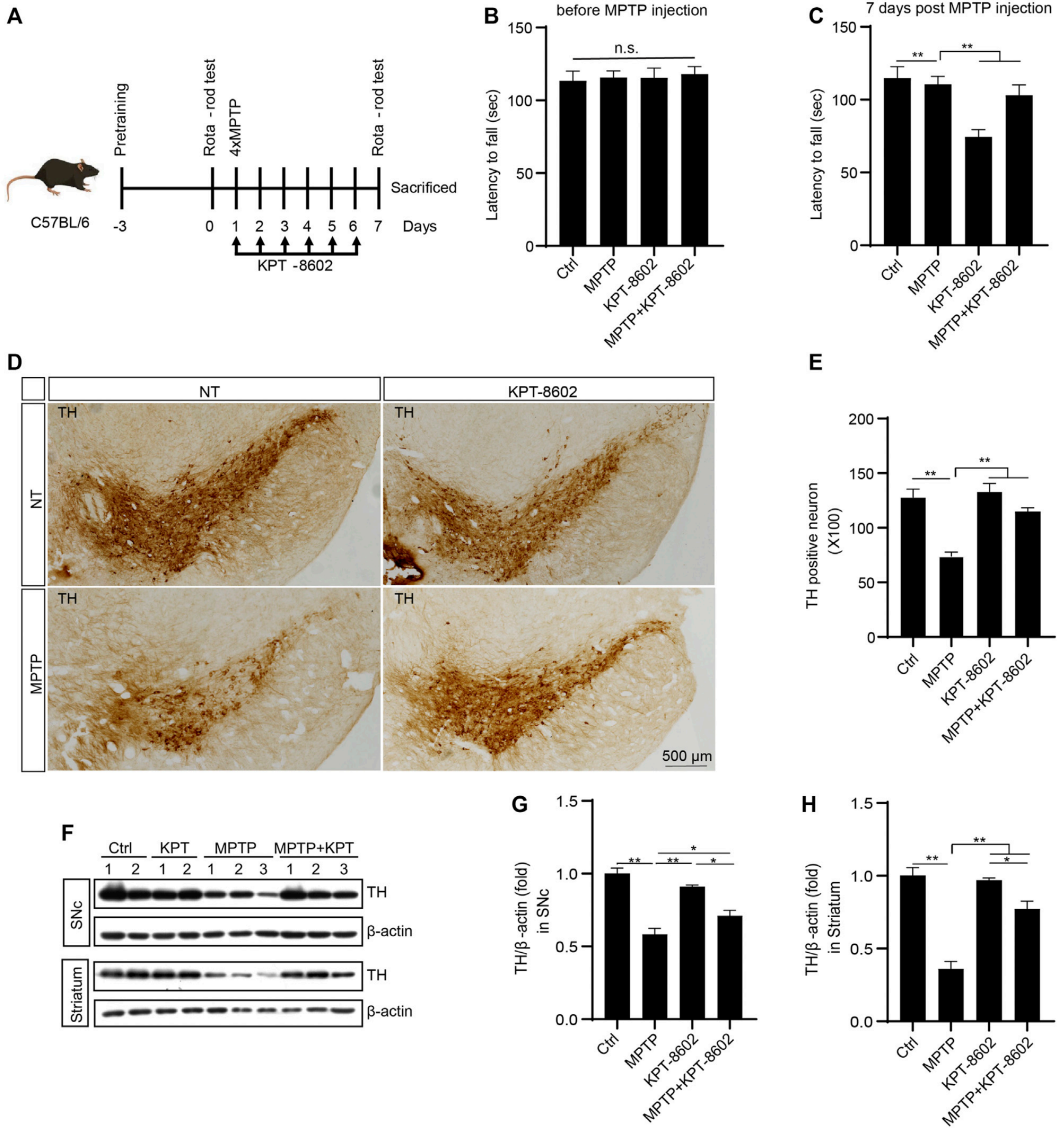
KPT-8602 ameliorates PD model mice severity. C57BL/6 mice were treated with saline or MPTP (four i.p. injections of 20 mg/kg at 2 h intervals). KPT8602 (5 mg/kg) or vehicle were administered daily (7 mice per group). **(A)** Schedule of the PD model establishment and administration of KPT-8602. **(B,C)** Latency of the indicated mice on the rotarod. **(D)** Representative photomicrographs of tyrosine hydroxylase (TH)-stained sections from the substantia nigra compacta (SNc) of mice brain. Scale bars, 500 μm for images, respectively. **(E)** Number of TH-positive neurons was analyzed by ImageJ. The expression of TH and *β*-actin protein was analyzed by Western blotting **(F)**, and the gray value of their bands in the SNc **(G)** and striatum **(H)** were analyzed by ImageJ. (^*^ indicates *p* < 0.05, ^**^ indicates *p* < 0.01 by one-way ANOVA).

In this section, we investigated the potential effect of KPT-8602 in inhibiting neuroinflammation and attenuating neurodegeneration in the MPTP-induced PD mouse model (Al-Bachari et al., 2020) (Figure 6A). In the rotarod test, KPT-8602 administration attenuated locomotor incoordination caused by MPTP (Figures 6B, C). KPT-8602 also significantly increased the number of tyrosine hydroxylase (TH)-positive cells in the substantia nigra compacta (SNc) (Figures 6D, E), and increased TH expression in the SNc and striatum (Figures 6F–H), suggesting KPT-8602 protected dopaminergic neurons against MPTP-induced cell death.

**FIGURE 7:**
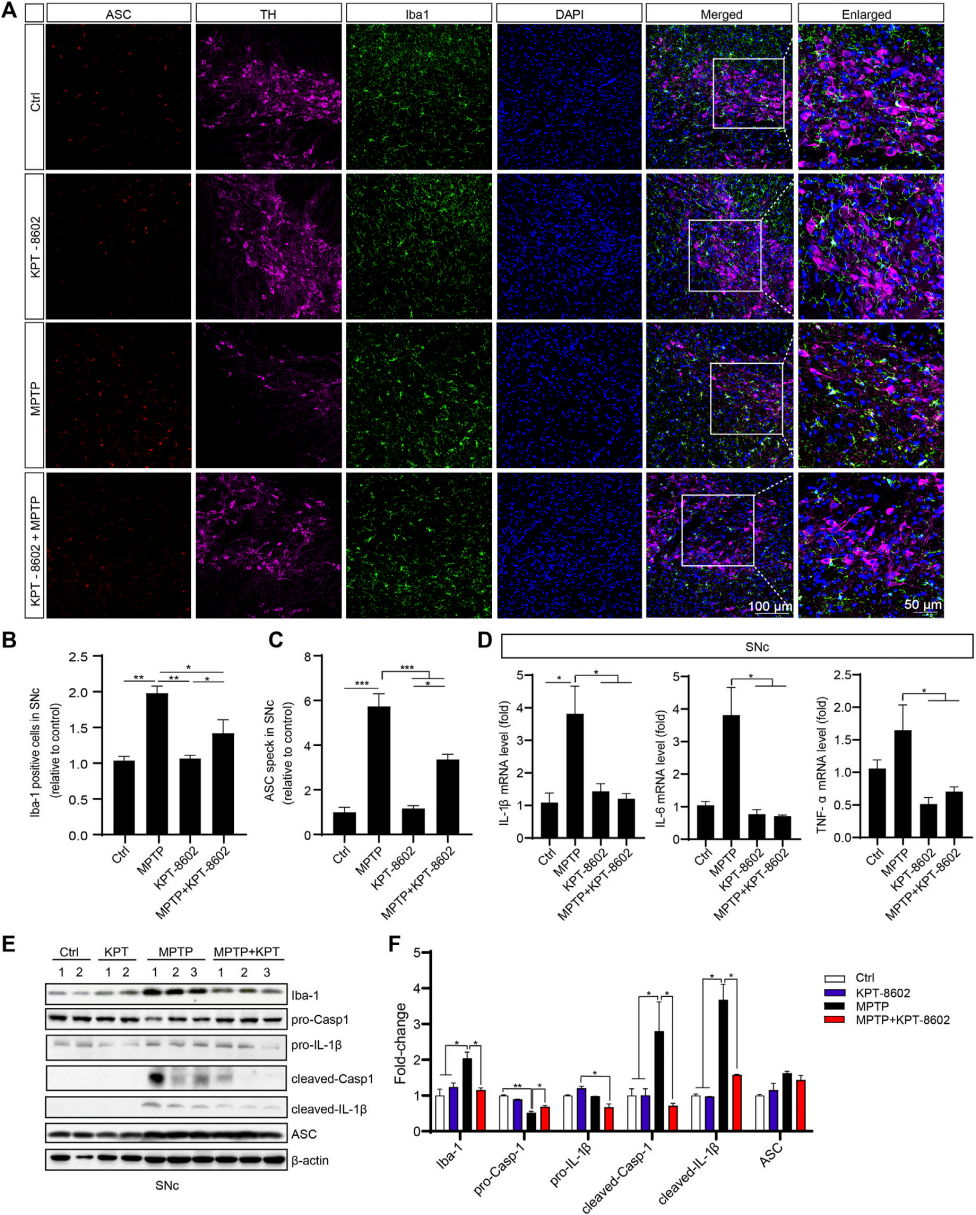
KPT-8602 inhibits activation of the NLRP3 inflammasome *in vivo*. **(A)** Substantia nigra compacta from PD mice administered KPT-8602 or vehicle were stained with an anti-Iba-1 and anti-ASC antibody to evaluate the activation of microglia and the NLRP3 inflammasome (5 mice per group). The number of Iba-1-positive cells **(B)** and ASC specks **(C)** was analyzed by ImageJ. Scale bars, 100 μm for low-magnification images and 50 μm for high-magnification images, respectively. **(D)** Expression levels of IL-1β, IL-6, and TNF-α in the SNc were detected by real-time PCR. The expression of Iba-1, pro-caspase-1, pro-IL-1β, cleaved caspase-1, cleaved-IL-1β, ASC, and *β*-actin in the SNc was analyzed by Western blotting **(E)**, and the gray value of their bands was analyzed by ImageJ **(F)**. (^*^ indicates *p* < 0.05, ^**^ indicates *p* < 0.01, ^***^ indicates *p* < 0.001 by one-way ANOVA).

To determine the role of microglia and the NLRP3 inflammasome activation in this process in the SNc, we co-stained TH and ASC with microglia indicated by ionized calcium-binding adaptor molecule 1 (Iba1). KPT-8602 administration caused a reduced number of amoeboid-like microglia (Figures 7A, B) and ASC speck (Figures 7A, C) in the SNc of the PD mice model. Consistent with this result, the expression of IL-1β, IL-6, and TNF-α in the SNc area was also reduced in the KPT-8602-treated PD mice model (Figure 7D). Consistently, MPTP-induced activation of caspase-1 cleavage and maturation of IL-1β were also ameliorated by KPT-8602 (Figures 7E, F). Taken together, these results demonstrated that KPT-8602 demonstrated neuroprotective effects against MPTP-induced dopaminergic neuron loss and the NLRP3 inflammasome activation *in vivo*.

## Discussion

In this study, we demonstrate that KPT-8602 exhibits a strong anti-inflammatory effect by blocking the NF-κB pathway and the NLRP3 inflammasome. More importantly, KPT-8602 had significant therapeutic effects on LPS-induced systemic inflammation and MPTP-induced PD in mice. This finding may provide new therapeutic approaches for NF-κB and NLRP3-driven diseases and suggests that KPT-8602 can be used to treat inflammatory diseases in addition to cancer.

Our results indicated that KPT-8602 was identified to block the activation of the NLRP3 inflammasome by inhibiting the NF-κB pathway. The NF-κB pathway is considered the “priming” signal for the NLRP3 inflammasome activation (Zheng et al., 2020). The classical activation of NF-κB is dependent on the phosphorylation of IκBα and p65, followed by degradation of IκBα and nuclear translocation of p65 (Fu et al., 2021). Here, we found that upon the stimulation with LPS, cells pretreated with KPT-8602 displayed decreased phosphorylation of IκBα and p65, especially the phosphorylation and translocation of p65. One possible mechanism is that KPT-8602 might inhibit the expression of XPO1 and thereby reduces the nuclear import of p65. It has been reported that inhibition of the nuclear exporting activity of XPO1 causes the accumulation of p65 in the nuclear (Kashyap et al., 2016). In our result, however, no accumulation of p65 in nuclear was observed in KPT-8602-treated cells and XPO1-silenced cells. This difference was potentially caused by different mechanisms of different inhibitors of XPO1. In addition, we found that cells treated with KPT-8602 displayed a lower level of phosphorylated IκBα, indicating that XPO1 was involved in the process of IKKα/β-mediated phosphorylation of IκBα. However, the mechanism of KPT-8602 in regulating inflammasome is unclear. It is well known that upregulation of NLPR3 and pro-IL-1β is the “priming” signal for the inflammasome activation (Sutterwala et al., 2014). Our results demonstrated that KPT-8602 could directly suppress the transcription of NLRP3 and pro-IL-1β, impeded the assembly of the NLRP3 inflammasome, and thereby decreased the release of mature cytokines.

Increasing evidence indicates that the activation of the NF-κB pathway and the NLRP3 inflammasome is widely involved in different diseases, such as colitis, diabetes, and COVID-19 infection-induced pneumonia in the peripheral tissue (Wang et al., 2018; An et al., 2019; Xian et al., 2021). Aberrant activation of the NLRP3 inflammasome is also involved in the development of several neurodegenerative disorders, such as PD, Alzheimer’s disease (AD), and multiple sclerosis (Heneka et al., 2013; Haque et al., 2020; Malhotra et al., 2020). The inhibition of NLRP3 inflammasomes ameliorates the clinical and pathological symptoms of these diseases (Martinon et al., 2006; Duewell et al., 2010; Heneka et al., 2013; Lee et al., 2013; Wang et al., 2019; Pan et al., 2021). However, only a few NLRP3-selective inhibitors are identified up to date, and most of these inhibitors are not available for clinical use (Coll et al., 2015; Jiang et al., 2017; He et al., 2018; Huang et al., 2018). Therefore, new drug development targeting the NF-κB signaling pathway and NLRP3 inflammasome potentially provides a new therapeutic avenue for the treatment of such diseases.

Herein, we demonstrated that KPT-8602 inhibited the activation of the NF-κB pathway and the NLRP3 inflammasome *in vitro*. Most importantly, KPT-8602 protected peripheral tissue injury against LPS-induced systemic inflammation and ameliorated CNS neuronal cell death and neuroinflammation induced by MPTP *in vivo*, suggesting that KPT-8602 was a promising candidate for the clinical treatment of inflammation-associated disease in the peripheral system and CNS. KPT-8602 is a second-generation XPO1-selective inhibitor, and recent studies suggest that XPO1 is involved in a variety of neurological and neuromuscular diseases (Hightower et al., 2020). Inhibition of XPO1 by KPT-350 increases the expression of neuroprotectant proteins and reduces the inflammatory response, leading to improved recovery of motor functions after TBI (Tajiri et al., 2016). Moreover, KPT-8602 is distinctly different from other XPO1 inhibitors due to its poor blood–brain barrier (BBB) permeability. This feature limits its toxicity and anti-inflammatory effects in the CNS. However, when the integrity of BBB is compromised due to diseases such as PD, AD, and stroke, the application of KPT-8602 in the treatment of such diseases is viable (Al-Bachari et al., 2020; Liao et al., 2020). Therefore, our results suggest that KPT-8602 can be used as a potential anti-inflammatory agent in the treatment of a variety of neurodegenerative diseases.

In conclusion, our study suggests that KPT-8602 could be used as an anti-inflammation agent by inhibiting the activation of the NF-κB pathway and the NLRP3 inflammasome. Mechanically, inhibition of XPO1 by KPT-8602 impaired IKKα/β-mediated phosphorylation of IκBα and thereafter decreased the nuclear translocation of p65. In addition, both LPS-induced peripheral inflammation and MPTP-induced neuroinflammation were attenuated by the administration of KPT-8602. Given the pivotal role of the NF-κB pathway and the NLRP3 inflammasome in the pathogenesis of PD, KPT-8602 was suggested to be a promising drug for the clinical treatment of such diseases.

## Data Availability

The original contributions presented in the study are included in the article/Supplementary Material, further inquiries can be directed to the corresponding authors.
