# Supplementary figures and images for: The XPO1 Inhibitor KPT-8602 Ameliorates Parkinson’s Disease by Inhibiting the NF-κB/NLRP3 Pathway
Source: Front Pharmacol. 2022 Jun 1;13:847605. doi: 10.3389/fphar.2022.847605 (PMC9200340; doi:10.3389/fphar.2022.847605)

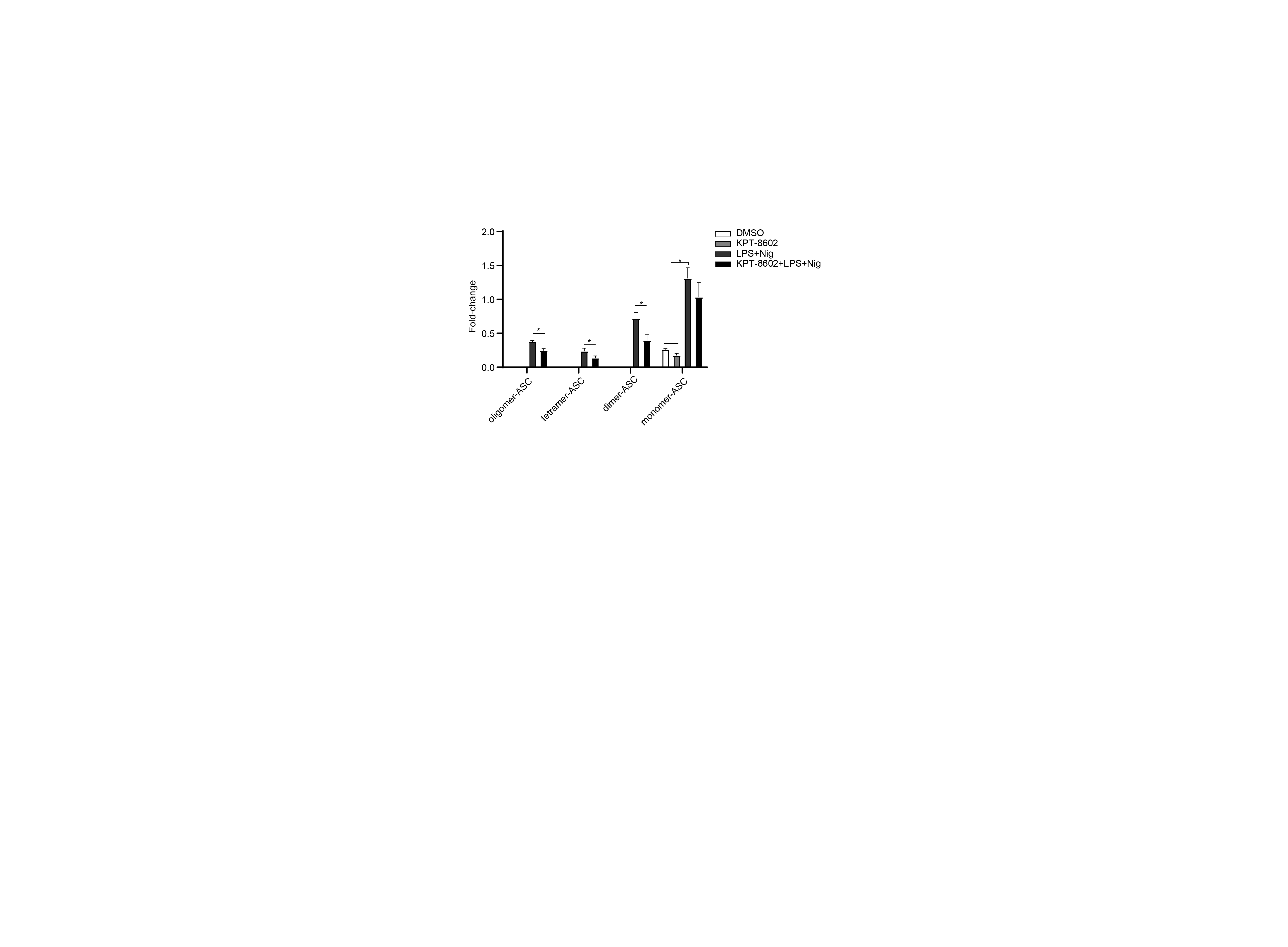

Supplement: Supplementary file 1 [file Image1.JPEG]

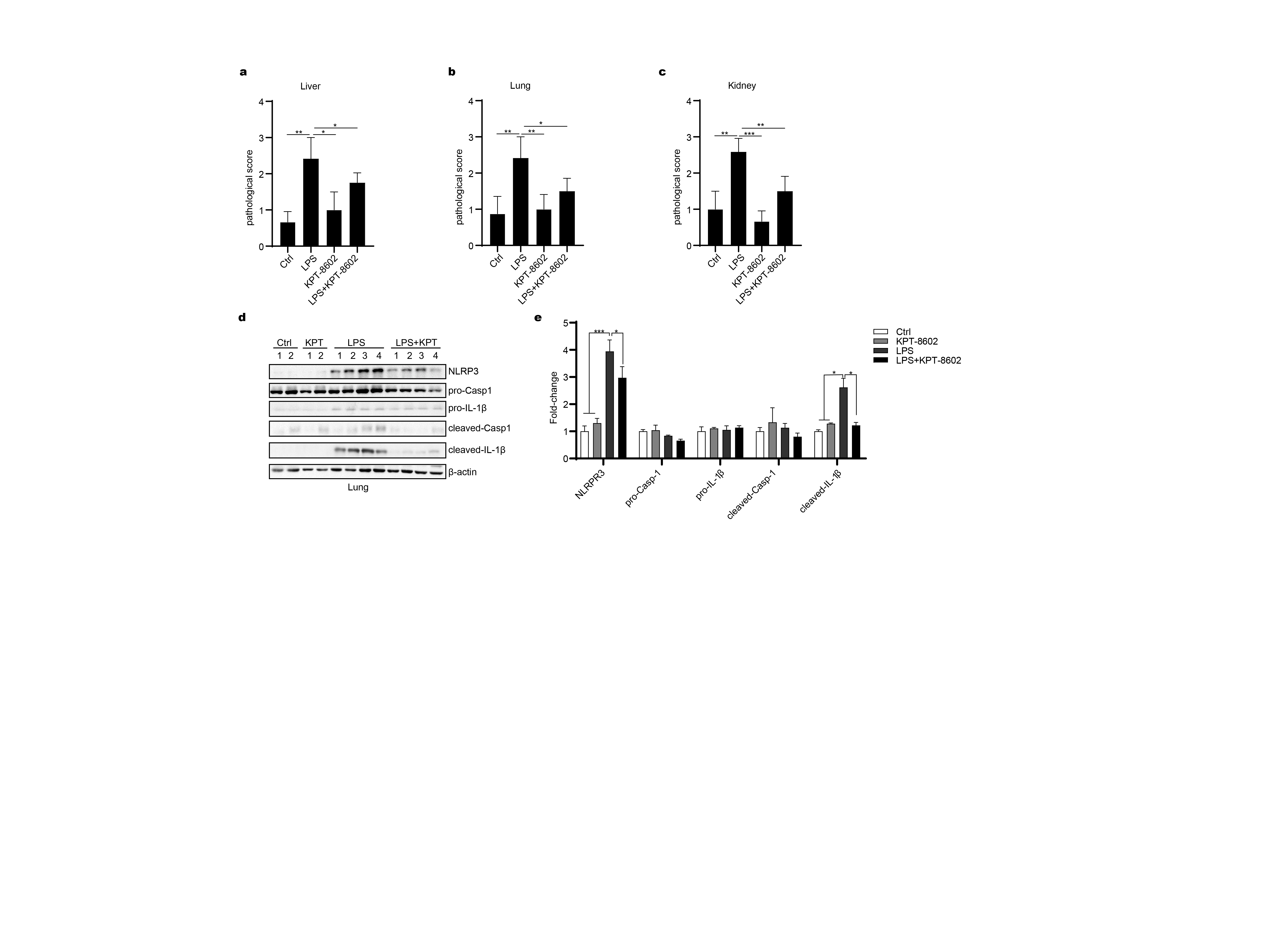

Supplement: Supplementary file 2 [file Image2.JPEG]
